# Inadequate Clearance of Translocated Bacterial Products in HIV-Infected Humanized Mice

**DOI:** 10.1371/journal.ppat.1000867

**Published:** 2010-04-29

**Authors:** Ursula Hofer, Erika Schlaepfer, Stefan Baenziger, Marc Nischang, Stephan Regenass, Reto Schwendener, Werner Kempf, David Nadal, Roberto F. Speck

**Affiliations:** 1 Division of Infectious Diseases and Hospital Epidemiology, University Hospital Zurich, Zurich, Switzerland; 2 Division of Clinical Immunology, University Hospital Zurich, Zurich, Switzerland; 3 Institute of Molecular Cancer Research, University Zurich, Zurich, Switzerland; 4 Kempf and Pfaltz Histological Diagnostics, Zurich, Switzerland; 5 Experimental Infectious Diseases and Cancer Research, Division of Infectious Diseases and Hospital Epidemiology, University Children's Hospital Zurich, Zurich, Switzerland; NIH/NIAID, United States of America

## Abstract

Bacterial translocation from the gut and subsequent immune activation are hallmarks of HIV infection and are thought to determine disease progression. Intestinal barrier integrity is impaired early in acute retroviral infection, but levels of plasma lipopolysaccharide (LPS), a marker of bacterial translocation, increase only later. We examined humanized mice infected with HIV to determine if disruption of the intestinal barrier alone is responsible for elevated levels of LPS and if bacterial translocation increases immune activation. Treating uninfected mice with dextran sodium sulfate (DSS) induced bacterial translocation, but did not result in elevated plasma LPS levels. DSS-induced translocation provoked LPS elevation only when phagocytic cells were depleted with clodronate liposomes (clodrolip). Macrophages of DSS-treated, HIV-negative mice phagocytosed more LPS ex vivo than those of control mice. In HIV-infected mice, however, LPS phagocytosis was insufficient to clear the translocated LPS. These conditions allowed higher levels of plasma LPS and CD8+ cell activation, which were associated with lower CD4+/CD8+ cell ratios and higher viral loads. LPS levels reflect both intestinal barrier and LPS clearance. Macrophages are essential in controlling systemic bacterial translocation, and this function might be hindered in chronic HIV infection.

## Author Summary

HIV infection leads to continuous destruction of the body's immune defenses. Furthermore, disease progression is linked to heightened levels of immune activation. However, the underlying activating factors and their relationships to HIV pathogenesis are controversial. In patients with chronic HIV infection, bacteria and their products, such as lipopolysaccharide (LPS), translocate from the intestinal lumen into the systemic circulation. In the current study, we investigated the pathogenic potential of bacterial translocation in HIV-infected humanized mice. By modulating the amount of bacterial translocation in the mice, we determined that LPS elevation depends on intestinal barrier dysfunction and defective LPS clearance by macrophages. HIV-infected mice showed inadequate LPS clearance, leading to a cascade of uncontrolled bacterial translocation, T-cell activation, HIV replication, and T-cell loss. Our study highlights how important the interplay between different immune cells is for maintaining a healthy balance between immune activation with the goal to defend the body against microbes and detrimental activation that fuels HIV replication.

## Introduction

The clinical course of HIV infection varies considerably among patients, and the variability is even greater in simian models. Asian monkeys infected with simian immunodeficiency virus (SIV) rapidly progress to AIDS, but African monkeys do not get sick [1]. In general, a pathogenic course of retroviral infection is characterized by high levels of immune activation [2], and bacterial translocation from the intestinal tract has been implicated as an underlying activating mechanism [3]–[8]. The integrity of the intestinal barrier is impaired early in acute retroviral infections [9], and a substantial fraction of the intestinal CD4+ T cells are lost within days after infection [10], [11].

However, bacterial translocation manifests itself only later. Low T-cell numbers in the gut are an important characteristic of HIV or SIV pathogenesis [12], [13], but intestinal CD4+ T-cell depletion does not predict the outcome of SIV infection in monkeys [14]. In SIV-infected African monkeys, for example, bacterial translocation is prevented despite low numbers of intestinal CD4+ T cells [15]. Thus, intestinal CD4+ T-cell depletion alone cannot explain bacterial translocation and the subsequent rise in plasma lipopolysaccharide (LPS) levels in chronic HIV infection [8]. Preferential depletion of Th17 cells is associated with disruption of the intestinal barrier in pathogenic retroviral infections [16], [17], but overall, the mechanism linking bacterial translocation and HIV pathogenesis is not fully understood.

Seeking evidence for this mechanism, we examined relationships among intestinal barrier integrity, microbial translocation, immune activation, and HIV replication in a mouse-model of HIV infection. In this model, RAG2^−/−^gamma_c_^−/−^ mice are transplanted with human cord-blood hematopoietic stem cells. A human lymphoid system develops [18], and the “humanized” mice can be infected with HIV [19]–[21]. The humanized mice combine the advantages of studying HIV in human cells and in a small-animal model that facilitates experimental manipulations. Furthermore, these mice have low intestinal lymphocyte numbers [22]. In HIV infection, low numbers result mostly from virus-mediated depletion. In the humanized mice, however, they result from a low local level of engraftment by human cells. Thus, in the humanized mice, low numbers of intestinal T cells are uncoupled from the effects of viral replication. Moreover, the intestinal barrier can be disrupted by adding dextran sodium sulfate (DSS) to the drinking water of mice [23]. DSS treatment leads to apoptosis [24] and reduced proliferation of intestinal epithelial cells [25], mimicking the enterocyte apoptosis seen in SIV infection [26].

In the current study, we dissected the effects of bacterial translocation alone or in the context of HIV infection by combining DSS and HIV in humanized mice. We defined the consequences of HIV infection and bacterial translocation on plasma LPS levels, LPS clearance by macrophages, immune activation, and T-cell loss.

## Results

### DSS treatment or HIV infection induce a similar amount of bacterial translocation

**Figure 1:**
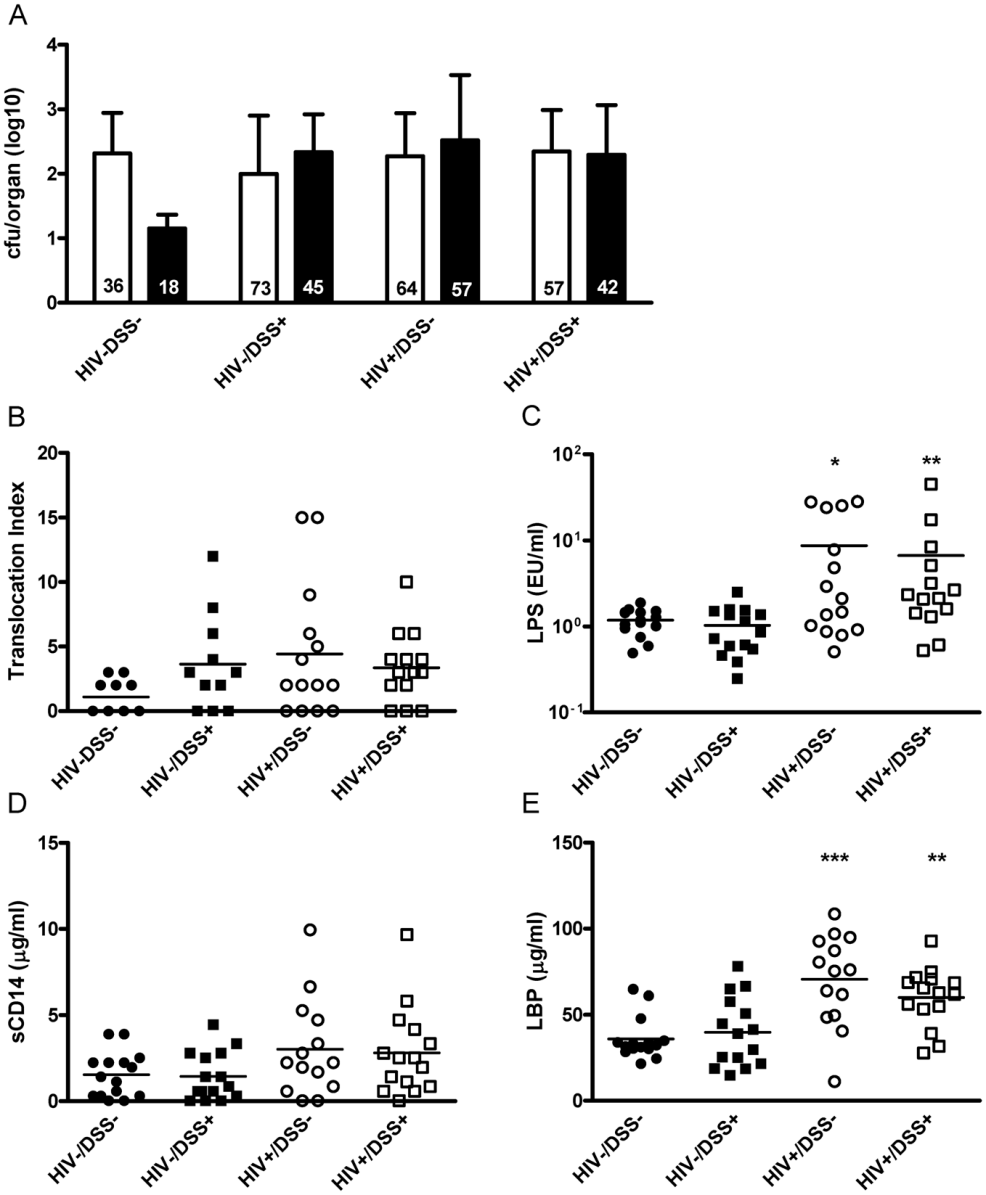
Gastrointestinal barrier dysfunction did not completely explain HIV-associated plasma LPS elevation in humanized mice. 4 to 6 weeks after HIV or mock infection, humanized mice received DSS 0.75% or normal drinking water for 2 weeks. (A) In cultures of organ suspensions, bacterial colony forming units from MLN (white bar) and spleens (black bar, mean, SD) were quantified; percentages of positive organs in the different groups are indicated at the bottom of the respective bars (n = 50, pooled data from two independent experiments). (B) The mice showed a trend towards higher levels of bacterial translocation (assessed by an index that includes number, species, and location of bacteria detected, P = 0.1, 0.08 and 0.13, respectively) after HIV infection or DSS treatment. (C) DSS+/HIV− mice (black square) had plasma LPS levels similar to those of control mice (black circle). Only HIV+ mice without (white circle, *, P = 0.015) or with DSS treatment (white square, **, P = 0.005) showed significant increases of plasma LPS (n = 59, pooled data from two independent experiments). (D and E) Both groups of HIV+ mice showed a trend towards higher plasma sC14 (P = 0.06 for HIV+/DSS− and P = 0.1 for HIV+/DSS+ mice) and had significantly higher LBP values (***, P = 0.0006 for HIV+/DSS− and **, P = 0.008 for HIV+/DSS+ mice).

In a DSS dose-response experiment, we established a treatment protocol that increases bacterial translocation without inducing colitis (Fig. S1). Briefly, we quantified bacterial translocation in groups of HIV-uninfected and -infected mice with mock or DSS treatment (i.e., HIV−/DSS−, HIV−/DSS+, HIV+/DSS−, or HIV+/DSS+ mice). We infected humanized mice with the CCR5-tropic HIV strain YU-2 and verified the infection by RT-PCR of plasma HIV RNA 4–6 weeks after inoculation. Thereafter, infected and uninfected control mice were treated with 0 or 0.75% DSS for 2 weeks. We cultured organ suspensions of mesenteric lymph nodes (MLN) and spleens, quantified bacterial colonies (Fig. 1A), and calculated a translocation index, based on quantity, diversity, and location of recovered bacteria (Fig. 1B). HIV−/DSS− control mice showed some baseline translocation with roughly a third of the animals containing bacteria in the MLN suspensions. Only few animals showed systemic dissemination to the spleen. DSS treatment increased bacterial translocation; in HIV−/DSS+ mice percentages of cultures containing bacteria doubled, and particularly in the spleen bacterial loads were higher. Organ cultures yielded mainly *Lactobacilli*, *Staphylococcus xylosus*, a typical mouse commensal, and *Enterococci*, whereas stool cultures yielded a multitude of aerobic and anaerobic bacteria. Only some bacterial species translocated to the organs in sufficient numbers to be cultured. HIV+ mice had similar microbiology results to HIV−/DSS+ mice. Overall, the range and amount of translocation were comparable between HIV−/DSS+, HIV+/DSS−, and HIV+/DSS+ mice. HIV infection alone seemed to facilitate bacterial invasion from the gut, and DSS treatment did not further increase the bacterial translocation in HIV+ humanized mice.

### Bacterial translocation does not always induce LPS elevation

In humans bacterial translocation is quantified by measuring surrogate markers, such as plasma LPS, soluble CD14 (sCD14), or LPS binding protein (LBP). We compared these markers to the direct measurement of intestinal barrier function in humanized mice.

Plasma LPS measurements showed a contrasting picture to microbiology results. Only HIV+ mice exhibited elevated levels of LPS in the systemic circulation. HIV−/DSS+ mice, which had increased intestinal permeability according to the organ culture results, controlled plasma LPS levels (Fig. 1C). In accordance with the elevated LPS levels, HIV+ mice also exhibited higher plasma sCD14 and LBP levels (Fig. 1D and E). We did not measure endotoxin core antibodies (EndoCAb) since humanized RAG2^−/−^gamma_c_^−/−^ mice in general have very poor antibody responses, serum immunoglobulin concentrations are several log lower than in humans [18], and mostly IgM is produced with IgG appearing only several months after humanization [20].

The HIV+ mice might have had a greater influx of smaller bacterial products and as a consequence higher plasma LPS levels. To eliminate this possibility, we measured the integrity of the intestinal barrier by gavaging mice with fluorescein isothiocyanate (FITC)-dextran with a molecular weight similar to that of LPS (Fig. S2B). Furthermore, we included wild-type and non-humanized mice to determine the consequences of irradiation and transplantation. After 4 hours, FITC-dextran was detected at similar concentrations in the plasma of HIV−/DSS− control mice, wild-type BALB/c, and non-humanized RAG2^−/−^gamma_c_^−/−^ mice. Thus, independent of humanization, RAG2^−/−^gamma_c_^−/−^ and wild-type mice had equal intestinal permeabilities, and the absence of intestinal lymphocytes had little effect on permeability in this model. In DSS-treated or HIV-infected mice there was a trend towards higher FITC-dextran values. In histological sections of the intestines, we found no evidence for exacerbated damage in HIV+ mice, and DSS-treated mice showed moderate changes (Fig. S2A).

Defects in intestinal barrier integrity influenced translocation, but alone they were not sufficient to induce LPS elevation. In HIV-infected mice, some additional factors contributed to higher plasma LPS levels.

### Bacterial translocation can be compensated for by increased macrophage phagocytosis

**Figure 2:**
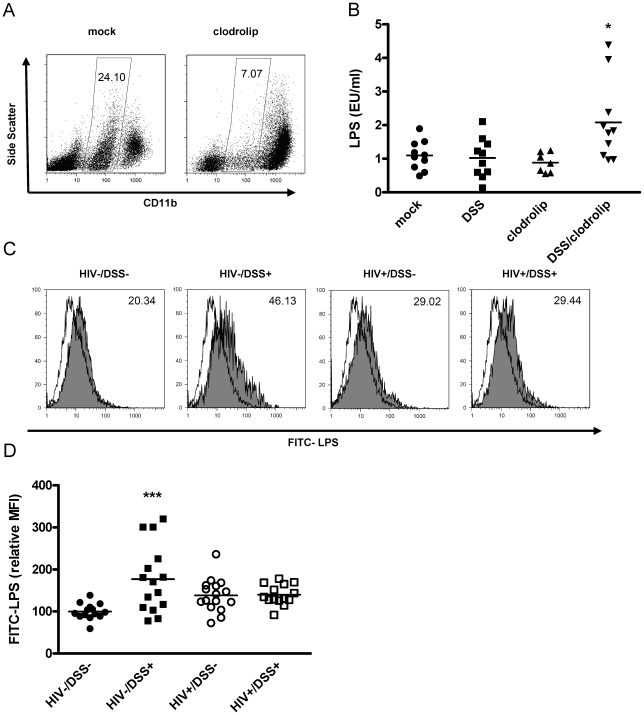
The combination of bacterial translocation and disturbed LPS clearance induced plasma LPS elevation. (A) Humanized mice were injected intraperitoneally with clodrolip (1 mg/20 g body weight) to deplete phagocytic CD11b intermediate cells (spleens of representative mock PBS or clodrolip treated mice 48 h after injection). (B) After 1 week of DSS 0.75% (square, block arrow down) treatment and a second injection of clodrolip (0.5 mg/20 g body weight) (block arrow up, block arrow down), plasma LPS was only increased in mice that received both treatments (*, P = 0.006, n = 37, pooled data from two independent experiments). (C) Liver macrophages, isolated from HIV− or HIV+ mice that received either normal drinking water or 0.75% DSS for 2 weeks, were incubated ex vivo with FITC-LPS at 37°C or 4°C (shaded or open histogram), and mean fluorescence intensity of phagocytic cells (values upper right corner) was measured. (D) Values were normalized to the mean FITC-LPS signal of cells from HIV−/DSS− mice (black circle). DSS-induced bacterial translocation increased FITC-LPS phagocytosis (black square, ***, P<0.0001), but HIV infection abrogated this effect (white circle, P = 0.2), independent of DSS treatment (white square, P = 0.19) (n = 59, pooled data from two independent experiments).

We hypothesized that plasma LPS levels are a marker for bacterial translocation and for the clearance of bacterial products from the systemic circulation. One of the main LPS clearance mechanisms is phagocytosis by liver macrophages [27], [28]. We tested the influence of LPS clearance on plasma LPS levels by depleting macrophages in the humanized mice. Clodronate liposomes induce apoptosis of phagocytic cells, thus humanized mice injected with clodronate liposomes exhibited a strong reduction of macrophage numbers (Fig. 2A). When we simultaneously treated the mice with 0.75% DSS for 1 week, plasma LPS levels increased (Fig. 2B). Disturbing the intestinal barrier or reducing the number of macrophages alone caused no change in plasma LPS.

To assess macrophage function during HIV infection, we isolated liver macrophages from all four groups of mice (i.e., HIV−/DSS−, HIV−/DSS+, HIV+/DSS−, and HIV+/DSS+ mice) and incubated the cells ex vivo with FITC-LPS (Fig. 2C). All macrophages took up some LPS, but cells isolated from HIV−/DSS+ mice up-regulated their phagocytic capacity significantly compared to cells from control animals. Cells from HIV+ mice showed a slight, statistically insignificant increase of LPS phagocytosis (Fig. 2D). Further evidence of altered macrophage function in HIV+ mice was an increase of pro-inflammatory cytokines, such as IL-12 and TNF-alpha in the plasma (Fig. S3). Macrophage numbers in liver and intestines were similar in all groups (data not shown). The results imply that bacterial translocation and LPS influx can be compensated for by increased LPS phagocytosis and that this function was disturbed in HIV-infected mice.

### Bacterial translocation activates T cells

**Figure 3:**
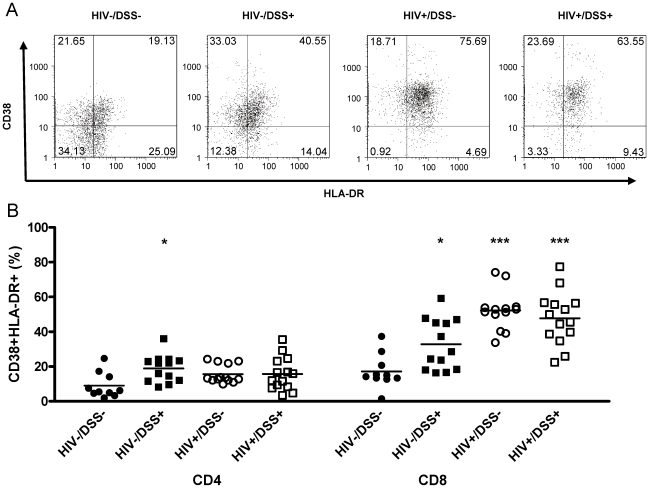
HIV-infected humanized mice had high levels of CD8+ T-cell activation. Control (black symbols) or HIV-infected mice (white symbols) received normal drinking water (circles) or 0.75% DSS (squares) for 2 weeks. (A) Activation levels in the spleen were determined by flow cytometry of human HLA-DR and CD38 staining of CD45+CD8+ (one representative animal per group) and CD45+CD4+ splenocytes. (B) DSS treatment alone slightly increased activation levels of CD4+ (*, P = 0.004) and CD8+ cells (*, P = 0.007), over those in uninfected, untreated control mice. HIV infection drastically increased CD8+ cell activation in animals that received normal (***, P<0.0001) or DSS (***, P<0.0001) water (n = 50, pooled data from two independent experiments).

Since bacterial translocation has been implicated in immune activation during chronic HIV infection, we examined the effects of bacterial translocation alone or in the context of HIV infection on the expression of cell-surface markers of T-cell activation. We determined T-cell activation levels in the spleens of humanized mice by measuring percentages of CD38 HLA-DR double positive cells in human CD4+ or CD8+ cells (Fig. 3A). CD4+ cells from HIV−/DSS+ animals showed a slight increase of activation, but activation of CD8+ cells was more apparent (Fig. 3B). Both groups of HIV-infected mice had even higher CD8+ cell activation levels, with no difference between DSS− and DSS+ groups. There was a trend towards slightly higher CD4+ cell activation in HIV+ mice.

Thus, bacterial translocation, even if no detectable plasma LPS elevation occurred, activated CD4+ and CD8+ cells in HIV- mice. In HIV+ mice, where levels of plasma LPS were increased, CD8+ cell activation was even stronger.

### T-cell activation promotes viral replication and CD4+ T-cell loss

**Figure 4:**
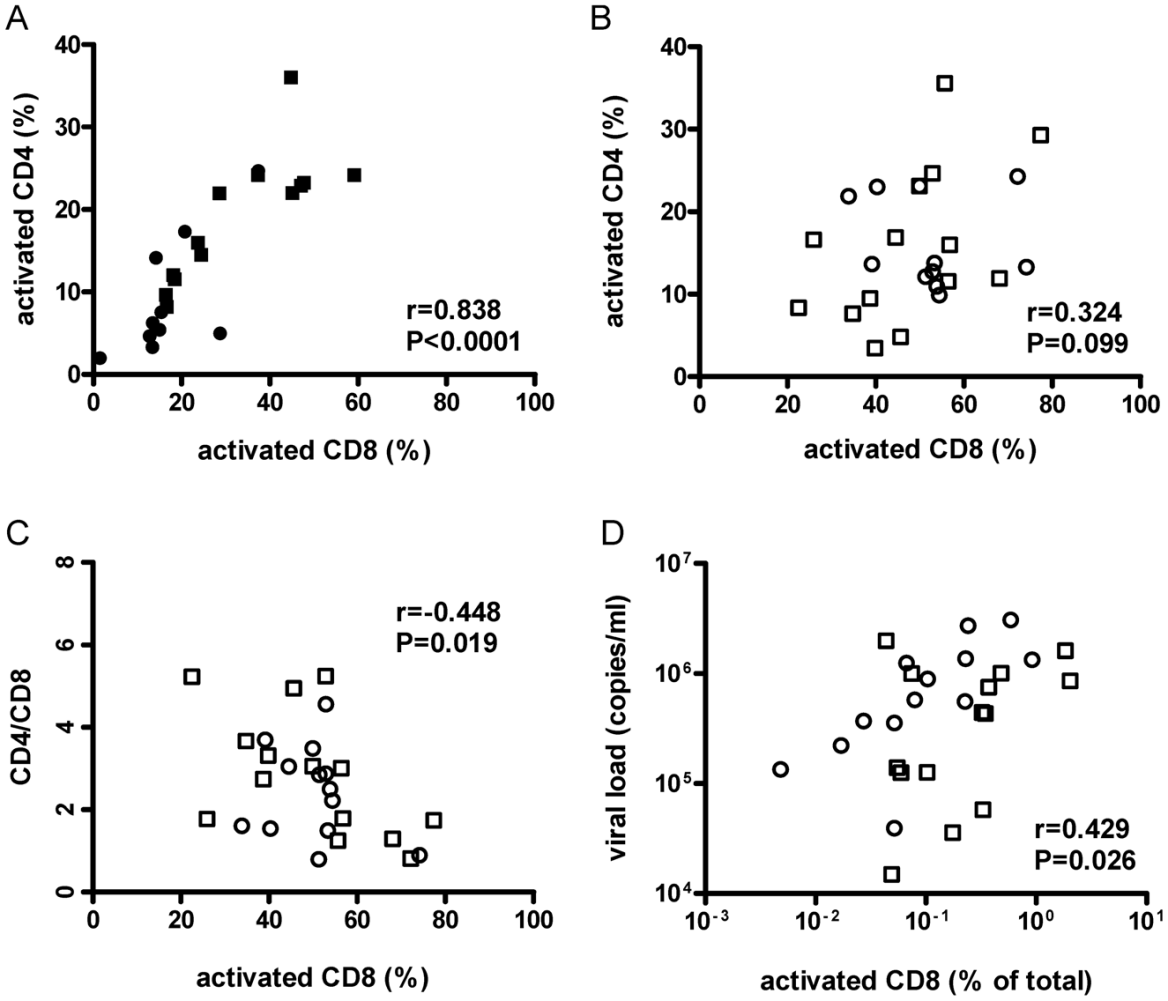
Activation of CD8+ cells was associated with lower CD4+/CD8+ cell ratios and higher viral loads. Human CD8+ splenocyte activation levels defined by HLA−DR+ and CD38+ co-staining from HIV−/DSS− (black circle), HIV−/DSS+ (black square), HIV+/DSS− (white circle), and HIV+/DSS+ animals (white square) were correlated with CD4+ cell-activation levels, CD4+/CD8+ cell ratio, and viral load. (A) In uninfected mice, activation levels of CD8+ and CD4+ cells were tightly correlated (n = 23, pooled data from two independent experiments). (B) But in HIV+ mice, this relationship was not as clear (n = 27, pooled data from two independent experiments). (C) In HIV+ mice, higher percentages of activated CD8+ cells correlated with lower CD4+/CD8+ cell ratios. (D) When activation levels were adjusted for overall engraftment by calculating percentages of HLA-DR+CD38+CD8+ cells of total cells, including murine cells, CD8+ cell activation correlated also with higher viral loads.

In HIV- mice, levels of CD4+ and CD8+ cell activation were tightly correlated (Fig. 4A). If that relationship of CD4+ and CD8+ cell activation was the same in HIV+ mice, then CD4+ cell activation levels should have been even higher in HIV+ mice than in HIV−/DSS+ mice. The correlation between activation levels of CD4+ and CD8+ cells in HIV+ mice, however, was partially lost (Fig. 4B), and the HIV+ mice had a relative deficit of activated CD4+ cells.

In humanized mice, absolute CD4+ T-cell numbers differ, because the efficiency of human engraftment is variable. However, CD4+/CD8+ cell ratios are independent of engraftment and are, thus, reasonably reliable estimates of CD4+ cell depletion. The ratios were similar for all four groups (Fig. S4A). Individual variations of CD4+/CD8+ cell ratios between mice were probably too large to detect small changes between groups in the relatively short time of the experiment. Furthermore, no difference in HIV replication was observed between HIV+/DSS− and HIV+/DSS+ mice (Fig. S4B). Nevertheless, in HIV+ mice, percentages of activated CD8+ cells correlated with lower CD4+/CD8+ cell ratios (Fig. 4C). Hence, HIV+ mice with high levels of CD8+ cell activation lost more CD4+ cells. No correlation between CD4+/CD8+ cell ratios and CD8+ cell activation was seen in HIV− mice (data not shown, P = 0.423, r = 0.190). In the absence of HIV infection, no loss of CD4+ cells occurred, indicating that HIV causes the preferential loss of activated CD4+ cells. Indeed, higher CD8+ cell activation levels—this time calculated in relation to total splenocytes to take engraftment level also into account—correlated with higher viral loads (Fig. 4D). In the HIV+ humanized mice, activation of CD4+ cells seemed to be masked by HIV infection and rapid loss of these cells, maybe even before full expression of activation markers.

## Discussion

Dysfunction of the intestinal barrier has severe consequences for the whole organism. It leads to translocation of bacteria from the gut and mediates inflammation. In chronic HIV infection, for instance, elevated levels of circulating bacterial products are associated with T-cell activation and disease progression [8]. We established a humanized mouse model of intestinal barrier dysfunction to determine the systemic effects of bacterial translocation. In our model, permeability corresponded well with translocation. But plasma LPS levels, the classical marker of bacterial translocation, depended only partially on barrier dysfunction. Macrophages compensated for the increased bacterial translocation by up-regulating their phagocytic capacity and thereby kept plasma LPS levels in a normal range. In HIV-infected mice, however, LPS clearance was inadequate leading to increased plasma LPS levels, high T-cell activation, and vigorous HIV replication.

A multilayered barrier protects the body from invading intestinal bacteria. The first line of defense is a tight, mucus-coated sheath of intestinal epithelial cells. Moreover, leukocytes in the underlying lamina propria contribute to the protection against bacteria. The humanized mice we used have low intestinal lymphocytes numbers [22]. Therefore, changes of the epithelial integrity probably have a relatively big impact on translocation in humanized mice, even without obvious histopathological changes. While there was no obvious difference in FITC-dextran translocation between humanized and wild-type mice, intestinal permeability tended to increase after HIV infection or low dose DSS treatment (Fig. S2B). Notably, HIV+ mice had high plasma TNF-alpha levels (Fig. S3). TNF-alpha disrupts tight junctions and induces apoptosis of intestinal epithelial cells [29], thereby mediating barrier dysfunction. Baseline translocation in HIV−/DSS− mice was quite high with one third of the MLN cultures containing bacteria (Fig. 1A and B). HIV+ or DSS+ mice more frequently had bacteria translocating to MLN and spleen—in accordance with the increased FITC-dextran permeability. From our data, it is not possible to infer the exact role disturbance of epithelial permeability and intestinal lymphocyte depletion plays in human HIV infection. But in general, the amount of bacterial translocation depends on barrier integrity.

Surprisingly, plasma LPS levels showed a different pattern (Fig. 1C). They did not depend strictly on permeability and translocation. In our experiments, HIV+ mice had elevated plasma LPS levels. HIV−/DSS+ mice controlled the increased influx of bacteria from the gut by raising their ability to clear LPS. LPS elevation only occurred in animals that had disturbance of the intestinal barrier and at the same time LPS clearance defects, either because of macrophage depletion or HIV infection (Fig. 2). Thus, macrophage phagocytosis seems to be critical in protecting against systemic translocation and failed clearance leads to systemic elevation of bacterial products. This hypothesis is supported by studies of inflammatory bowel disease. Plasma LPS levels are elevated in active inflammatory bowel disease, when the intestinal barrier is disrupted [4]. Furthermore, patients with Crohn's disease clear subcutaneously injected bacteria very slowly, their macrophages secrete few pro-inflammatory cytokines in response to bacteria or TLR ligands, and the transcription profiles of these macrophages show defects in vesicle trafficking and cytoskeletal organization [30]. This indicates that defects similar to the dysfunctional phagocytosis seen in our model might be important in inflammatory bowel diseases.

The mechanism leading to macrophage dysfunction in HIV+ mice is not clear. It is tempting to postulate induction of an “endotoxin-tolerant” macrophage phenotype in HIV−/DSS+ mice, and loss of tolerance induction in HIV+ mice. Endotoxin tolerance is characterized by programming of macrophages towards phagocytosis instead of production of pro-inflammatory cytokines upon LPS re-exposure [31]. Indeed, plasma IL-12 and TNF-alpha levels were normal in HIV−/DSS+ mice despite increased bacterial translocation. In contrast, HIV+ mice had high levels of pro-inflammatory cytokines (Fig. S3). At the moment, the factors inhibiting tolerance induction are unknown. Duration of translocation might play a role: HIV−/DSS+ mice had barrier dysfunction for the relatively short period of two weeks. Control of longer lasting translocation might not be as easy. Otherwise, the pro-inflammatory state generated by HIV infection might influence macrophage function. Macrophages integrate a broad range of environmental information. Some cytokines [32] and bacterial products [33], [34] sensitize cells to LPS stimulation. Furthermore, viral products and/or cytokines produced due to virus infection interfere with endotoxin tolerance induction in chronically HCV- [35] or HIV- [36] infected patients. Instead monocytes from these patients produce more TNF-alpha upon LPS re-stimulation.

So far, it is unknown if LPS clearance dysfunction also exists in HIV-positive humans, although some evidence supports the existence of macrophage defects in chronic HIV infection. Plasma LPS levels in acutely HIV-infected patients are not elevated [8], despite early depletion of gut lymphocytes. During treatment interruption, LPS levels rise only after a few weeks of viral replication [7]. Early after antiretroviral therapy is stopped, falling EndoCAb levels indicate functional LPS clearance. Thereafter, elevation of LPS levels suggests the onset of clearance defects. In fact, HIV inhibits macrophage maturation [37] and phagocytosis [38], [39]. Monocytes from HIV-infected individuals show impaired *Mycobacterium* phagocytosis [40]; *Saccharomyces* up-take is also decreased, and LPS-mediated enhancement of phagocytosis is less than the enhancement in monocytes from healthy donors [41]. Moreover, monocytes from HIV-infected patients show an attenuated pro-inflammatory cytokine response to LPS stimulation ex vivo [8], [42], maybe due to in vivo pre-stimulation. Serum IL-12 and TNF-alpha levels, which reflect in vivo cytokine production, are higher [43]–[46]- similar to the increased cytokine levels we observed in HIV+ mice. Overall, our findings in HIV-infected humanized mice resemble the results from HIV-infected humans.

Certainly, not all aspects of human HIV infection can be modeled accurately in humanized mice. For example, direct HIV infection of macrophages is rare in the mice. Engraftment of human monocytes was low; less than 2% of all monocytes were of human origin (data not shown). Most macrophages are of murine origin and therefore resistant to HIV infection. In humans, macrophage permissiveness to HIV infection varies from tissue to tissue. For example, intestinal macrophages are quite resistant to infection, but vaginal macrophages are readily infected [47]. However, productive infection of macrophages is, in general, infrequent in vivo [48], except for late stages of disease when opportunistic infections occur [49]. To definitely determine the importance of direct viral infection of macrophages, other models with bigger populations of human myeloid cells would be useful.

Lymphoid engraftment, however, is quite good in humanized mice. This allowed the investigation of T-cell activation in DSS-treated or HIV-infected mice. Bacterial translocation induced CD4+ and CD8+ cell activation (Fig. 4). Other activating factors (i.e., stimulation of TLR7/8 by HIV RNA [50]–[52]) might also have played a role. However, in HIV−/DSS+ mice, such other activating factors were not present, and bacterial translocation alone activated T cells. Since plasma LPS levels were not elevated in these mice, immune activation might have been mediated by other bacterial components, such as peptioglycan, flagellin, or bacterial DNA. Moreover, LPS flux—input from the gut and subsequent clearance—was greater in these mice. A greater LPS flux might activate the immune system. At least, it induced and sustained stimulation of macrophage phagocytosis. Uncontrolled bacterial translocation, as seen in HIV+ mice, might reasonably caused higher levels of lymphocyte activation. Not surprisingly, DSS treatment had no effect on T-cell activation in HIV+ mice. The bacterial translocation indices and LPS phagocytosis capacities were almost identical in HIV+/DSS− and HIV+/DSS+ groups, leading to similar LPS, LBP and sC14 levels and, therefore, to similar activation levels.

Our results might explain why levels of CD8+ T-cell activation are especially good markers of disease outcome [53], even though CD8+ T cells are not direct viral targets. In HIV-uninfected mice CD8+ and CD4+ cell activation levels were tightly correlated. Thus, CD8+ T-cell activation levels seem to predict the amount of activated and HIV-permissive CD4+ T cells. These cells have a very short lifespan; the half-life of an infected CD4+ T cell is estimated at 1.6 days [54]. Additionally, measurements of lymphocyte telomere lengths indicate that, in HIV-infected individuals, only CD8+ T-cell turnover is increased, while telomeres in CD4+ T cells show no excessive turnover [55]. CD4+ T cells might be lost due to HIV infection before cell division is accomplished. This could also explain the relative deficit of activated CD4+ cells we observed in HIV+ animals. HIV preferably infects activated CD4+ cells [56]. Indeed, those mice with the highest percentages of activated CD8+ cells had the lowest CD4+/CD8+ cell ratios and the highest HIV plasma loads.

In conclusion, we identified a critical role of macrophages in protection from systemic bacterial translocation. In humanized mice, failure of LPS clearance was associated with high levels of T-cell activation and HIV replication. Macrophage dysfunction might be an underestimated mechanism in HIV-induced immunodeficiency and certainly warrants further investigation.

## Materials and Methods

### Ethics statement

All experiments were approved by ethical committees of the University Zurich and the Federal Veterinary Department and were conducted according to local guidelines (TschV, Zurich) and the Swiss animal protection law (TschG). Cord blood was collected with written consent of the parents.

### Generation and HIV infection of humanized mice

Mice were reconstituted and infected as described [19]. Briefly, newborn RAG2^−/−^gamma_c_^−/−^ mice were irradiated with 2×2 Gy. CD34+ cells were isolated from human cord blood with immunomagnetic beads (Miltenyi Biotec), and 2.75±0.5×10^5^ cells were transplanted into each mouse. After 10 to 16 weeks, the degree of blood engraftment was determined by flow cytometry of peripheral blood mononuclear cells stained for the panhuman marker CD45 in all mice (mean human cells/live cells 5.6±5.4% SD). Mice were infected intraperitoneally with HIV YU-2, 2×10^5^ of the tissue-culture infectious dose_50_ per mouse. Plasma viral loads were measured by RT-PCR (Amplicor, Roche) 4–6 weeks after inoculation and at the end of each experiment. The detection limit was 1,600 HIV RNA copies/ml. Activation levels of T cells were measured by flow cytometry of splenocytes stained for human CD45, CD4, CD8, HLA-DR, CD38, and appropriate isotype controls (all from BD Biosciences). In all experiments, mouse litters and cord blood donors were evenly distributed to all experimental groups.

### Induction and measurement of bacterial translocation

Bacterial translocation was induced by adding 0.75% (w/v) DSS (molecular weight 40,000, MP Biomedicals) to the drinking water for 2 weeks. Spleen and MLN were removed aseptically, mashed with a pestle in PBS, and plated on sheep blood and MacConkey agar plates. Plates were only incubated aerobically, since in a pilot experiment, no anaerobic bacteria could be detected in organ cultures. As a control, diluted stool samples were cultured both in aerobic and anaerobic conditions. A descriptive bacterial translocation index was calculated from organ culture results (no bacterial growth  = 0 points, 10 to 99 cfu/organ  = 1 point, 100 to 999 cfu/organ  = 2 points, >1000 cfu/organ  = 3 points, for all organs points were multiplied by the number of bacterial species detected. For spleen cultures, points were doubled, and finally, all points from one mouse were added up). LPS was quantified by endpoint chromogenic limulus amoebocyte lysate assay (Lonza). Plasma samples were diluted 1∶10 with endotoxin free water, incubated at 85°C for 12 min, and assayed according to the manufacturer's instructions. Standard endotoxin (Lonza) was diluted to cover plasma LPS values within a range of 0.5 to 20 EU/ml. Plasma mouse sCD14 and LBP were measured by ELISA (both from CellSciences), according to the manufacturer's instructions. For sCD14 measurement, samples were diluted 1∶150, and for LBP measurement, 1∶800.

### Ex vivo LPS phagocytosis

Liver mononuclear cells were isolated by Ficoll-Hypaque density gradient centrifugation. To further purify macrophages, cells were incubated for 3 h in RPMI, 10% FCS, penicillin/streptomycin, and L-glutamine at 37°C, 5% CO_2_, and then washed two times with room temperature PBS to remove non-adherent cells. This procedure yielded over 90% murine CD11b+ cells. Cells were then incubated with 0.1 mg/ml FITC-LPS (Sigma) in RPMI, 10% FCS, penicillin/streptomycin, and L-glutamine at 37°C, 5% CO_2_ for 1 h. As a control, cells were also incubated with FITC-LPS at 4°C. Cells were then washed two times with cold PBS, detached with trypsin, washed once again, and analyzed by flow cytometry. FITC-LPS mean fluorescent intensity was normalized to the fluorescence of samples from HIV−/DSS− mice.

### Macrophage depletion

Mice were injected intraperitoneally with clodrolip (1 mg/20 g body weight) or with PBS [57]. After 48 h, depletion was verified in four mice by staining of spleen and liver cells for CD11b. To maintain depletion, mice were treated a second time with clodrolip 0.5 mg/20 g body weight or with PBS intraperitoneally 4 days after the first injection. Concurrently, half of the mice received DSS 0.75% (w/v) in their drinking water. After 1 week, the mice were sacrificed, and their plasma LPS levels were measured.

### Statistical analysis

GraphPad Prism 5.02 was used for statistical analysis. Data were analyzed by parametric one-way analysis of variance, followed by Bonferroni post-test. All p-values shown are adjusted for multiple comparisons. In the figures, p-values are presented for comparisons between treatment groups and controls and are denoted by asterisks. Values for HIV/DSS experiments for plasma LPS, viral load, FITC-dextran fluorescence, and percentages of activated CD8+ cells (of total cells) were log transformed before analysis to reduce right-skewing of the data. For all correlations, Pearson's correlation coefficient was calculated. In all figures, points represent values of individual mice, and lines depict mean values.

## Supporting Information

Figure S1 DSS induced bacterial translocation in humanized mice. 16 uninfected mice were treated with different doses of DSS in the drinking water for 2 weeks. (A) Systemic bacterial load was assessed by semi-quantitative (+ black bar, ++ white bar, +++) microbiological culture of organ suspension from mesenteric lymph nodes, spleen and liver. (B) Mouse weight as an indicator for diarrhea and colitis was measured daily (mean). This experiment was done once.
(0.32 MB TIF)

Figure S2 Histological and functional measurement of the intestinal integrity. (A) Formalin fixed, haematoxylin and eosin stained tissue sections of HIV infected and/or DSS treated mice showed moderate changes of the intestinal mucosa. DSS treatment induced villus blunting, a modest vessel dilation (*) and discreet goblet cell hyperplasia (>). (B) Humanized mice (n = 32, pooled data from two independent experiments) were infected with HIV (white symbols) or mock treated (black symbols) and 4 weeks later received 0 (circles) or 0.75% (squares) w/v DSS. After two weeks, in vivo permeability was measured by FITC-dextran (molecular weight 10,000, Sigma) translocation. Mice were gavaged with FITC-dextran 20 mg/20 g body weight in 200 µl PBS and four hours later FITC fluorescence in the plasma was measured. Wild-type (WT, block arrow up) and non-humanized RAG2^−/−^gamma_c_^−/−^ (RAG2, block arrow down) mice were included as a control. HIV−/DSS+ mice had higher FITC-dextran plasma values (*, P = 0.012) than WT, non-humanized and HIV−/DSS− control mice. Both HIV+ groups showed only a trend towards higher FITC-dextran translocation.
(3.69 MB TIF)

Figure S3 Macrophage derived pro-inflammatory cytokines in HIV+ mice. We measured plasma cytokine levels by cytometric bead assay (A.Urwyler, Cytolab) in HIV−/DSS− (black circle), HIV−/DSS+ (black square), HIV+/DSS− (white circle), and HIV+/DSS+ animals (white square). (A and B) Both human (left) and murine (right panel) cytokines were assessed. HIV+ mice showed a trend towards higher human (P = 0.084 and 0.096) and murine TNF-alpha levels (*, P = 0.045 and P = 0.11 for DSS− and DSS+ mice). (C and D) Human IL-12p40 was below detection limit in many of the animals, while murine IL-12p40 was significantly elevated in HIV+ mice (***, P = 0.0006, and **, 0.004 for DSS− and DSS+ mice). IL-1 beta was undetectable in all mice and IL-6 values showed no significant differences (data not shown).
(0.79 MB TIF)

Figure S4 Diversity of CD4+/CD8+ cell ratios and viral loads in humanized mice. Humanized mice were infected with HIV (white symbols) or mock treated (black symbols) and 4 weeks later received 0 (circles) or 0.75% (squares) w/v DSS. (A) After 2 weeks, spleens were removed and splenocytes were analyzed for human CD4+ and CD8+ T-cell ratios by flow cytometry (n = 50, pooled data from two independent experiments, no significant differences). (B) Plasma viral load was measured (n = 27, pooled data from two independent experiments), 48 h before the beginning of DSS treatment (baseline, no significant difference), and at the end of the experiment (after DSS, no significant difference).
(0.40 MB TIF)
