# Supplementary figures and images for: Inadequate Clearance of Translocated Bacterial Products in HIV-Infected Humanized Mice
Source: PLoS Pathog. 2010 Apr 29;6(4):e1000867. doi: 10.1371/journal.ppat.1000867 (PMC2861703; doi:10.1371/journal.ppat.1000867)

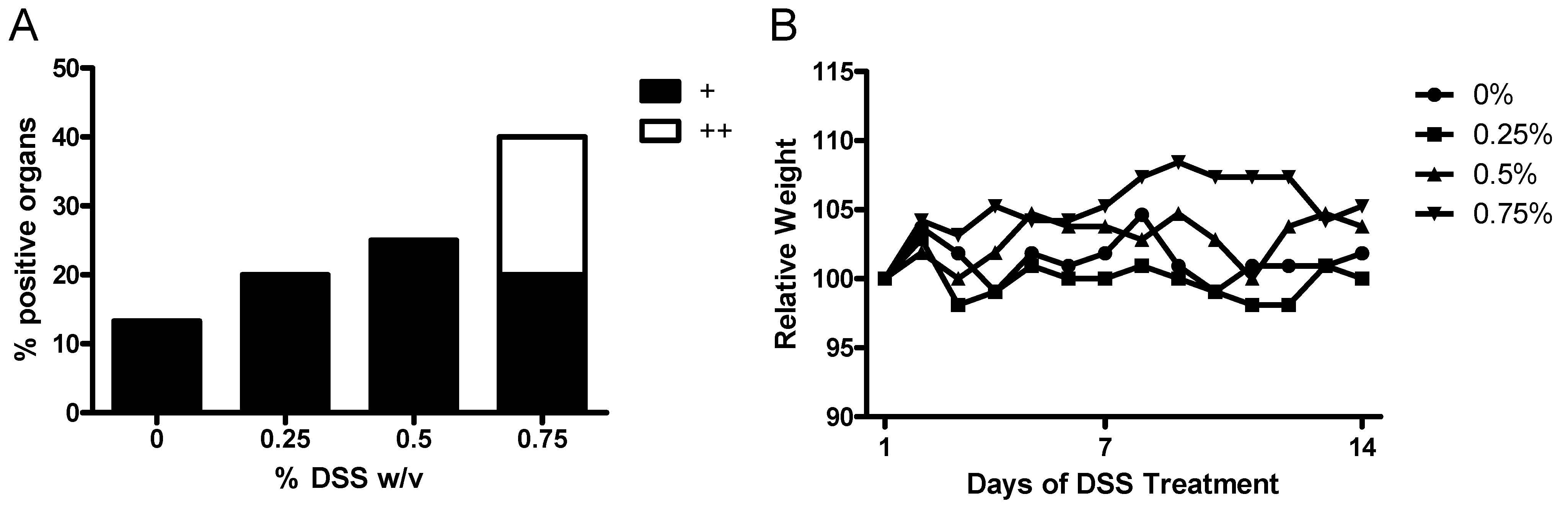

Supplement: Figure S1 — DSS induced bacterial translocation in humanized mice. 16 uninfected mice were treated with different doses of DSS in the drinking water for 2 weeks. (A) Systemic bacterial load was assessed by semi-quantitative (+ black bar, ++ white bar, +++) microbiological culture of organ suspension from mesenteric lymph nodes, spleen and liver. (B) Mouse weight as an indicator for diarrhea and colitis was measured daily (mean). This experiment was done once. (0.32 MB TIF) [file ppat.1000867.s001.tif]

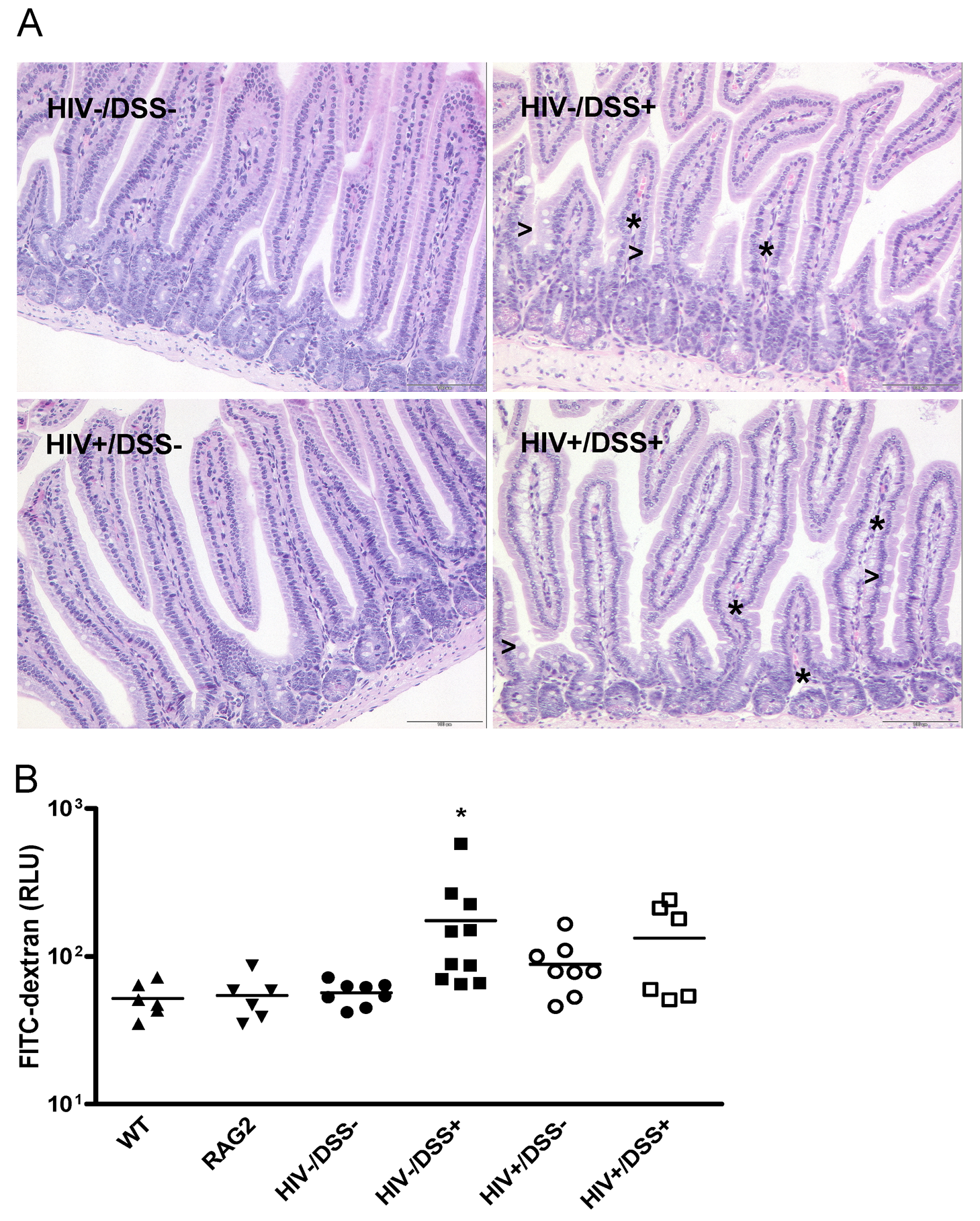

Supplement: Figure S2 — Histological and functional measurement of the intestinal integrity. (A) Formalin fixed, haematoxylin and eosin stained tissue sections of HIV infected and/or DSS treated mice showed moderate changes of the intestinal mucosa. DSS treatment induced villus blunting, a modest vessel dilation (*) and discreet goblet cell hyperplasia (>). (B) Humanized mice (n = 32, pooled data from two independent experiments) were infected with HIV (white symbols) or mock treated (black symbols) and 4 weeks later received 0 (circles) or 0.75% (squares) w/v DSS. After two weeks, in vivo permeability was measured by FITC-dextran (molecular weight 10,000, Sigma) translocation. Mice were gavaged with FITC-dextran 20 mg/20 g body weight in 200 µl PBS and four hours later FITC fluorescence in the plasma was measured. Wild-type (WT, block arrow up) and non-humanized RAG2−/−gammac −/− (RAG2, block arrow down) mice were included as a control. HIV−/DSS+ mice had higher FITC-dextran plasma values (*, P = 0.012) than WT, non-humanized and HIV−/DSS− control mice. Both HIV+ groups showed only a trend towards higher FITC-dextran translocation. (3.69 MB TIF) [file ppat.1000867.s002.tif]

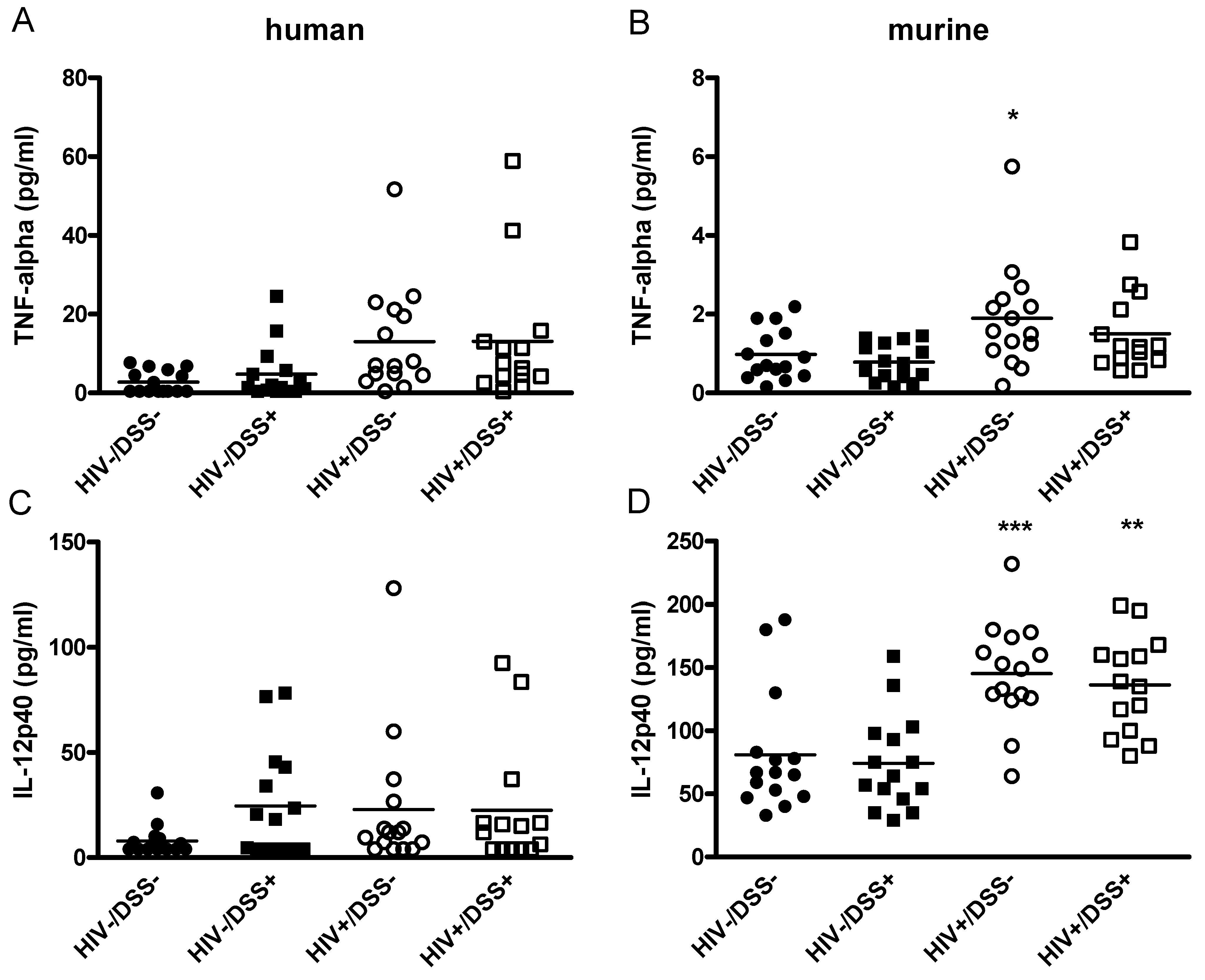

Supplement: Figure S3 — Macrophage derived pro-inflammatory cytokines in HIV+ mice. We measured plasma cytokine levels by cytometric bead assay (A.Urwyler, Cytolab) in HIV−/DSS− (black circle), HIV−/DSS+ (black square), HIV+/DSS− (white circle), and HIV+/DSS+ animals (white square). (A and B) Both human (left) and murine (right panel) cytokines were assessed. HIV+ mice showed a trend towards higher human (P = 0.084 and 0.096) and murine TNF-alpha levels (*, P = 0.045 and P = 0.11 for DSS− and DSS+ mice). (C and D) Human IL-12p40 was below detection limit in many of the animals, while murine IL-12p40 was significantly elevated in HIV+ mice (***, P = 0.0006, and **, 0.004 for DSS− and DSS+ mice). IL-1 beta was undetectable in all mice and IL-6 values showed no significant differences (data not shown). (0.79 MB TIF) [file ppat.1000867.s003.tif]

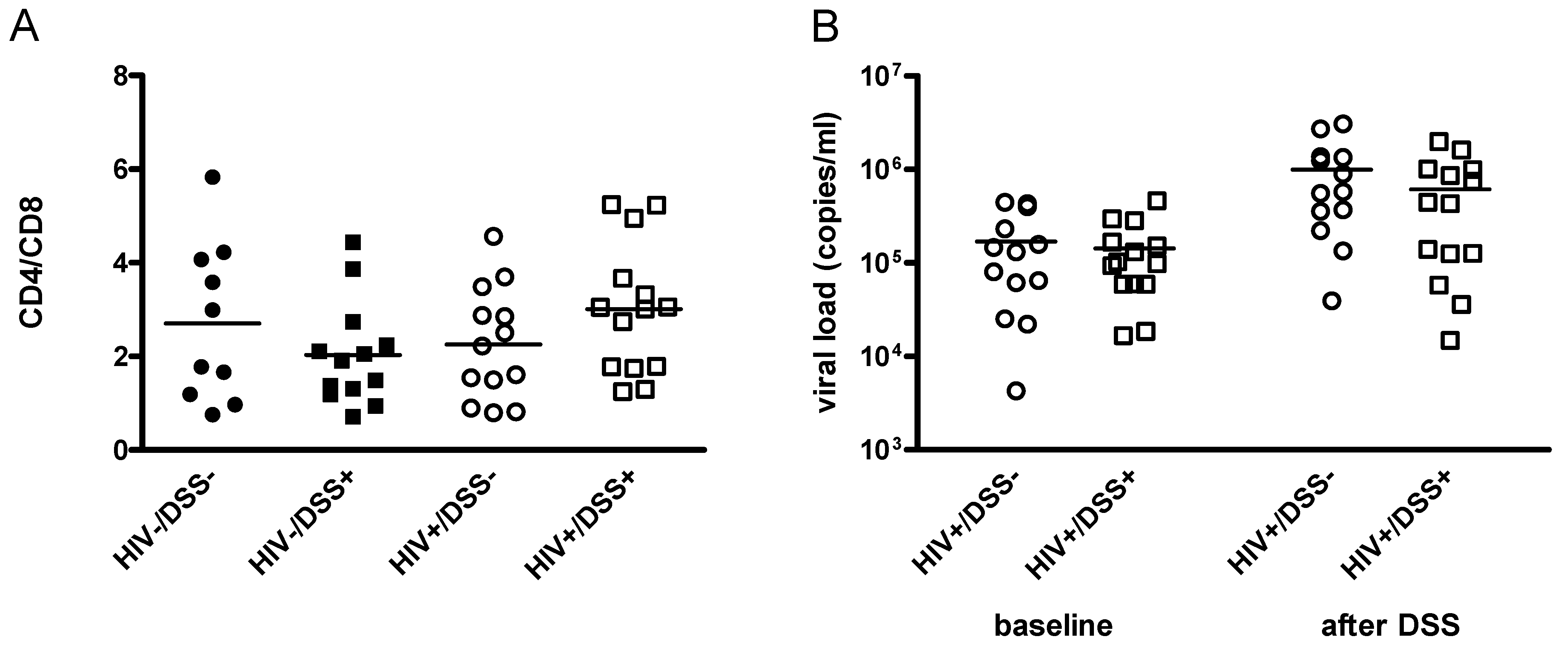

Supplement: Figure S4 — Diversity of CD4+/CD8+ cell ratios and viral loads in humanized mice. Humanized mice were infected with HIV (white symbols) or mock treated (black symbols) and 4 weeks later received 0 (circles) or 0.75% (squares) w/v DSS. (A) After 2 weeks, spleens were removed and splenocytes were analyzed for human CD4+ and CD8+ T-cell ratios by flow cytometry (n = 50, pooled data from two independent experiments, no significant differences). (B) Plasma viral load was measured (n = 27, pooled data from two independent experiments), 48 h before the beginning of DSS treatment (baseline, no significant difference), and at the end of the experiment (after DSS, no significant difference). (0.40 MB TIF) [file ppat.1000867.s004.tif]
